# Effect of undifferentiated versus hepatogenic partially differentiated mesenchymal stem cells on hepatic and cognitive functions in liver cirrhosis

**DOI:** 10.17179/excli2016-645

**Published:** 2016-11-07

**Authors:** Dalia Azmy Elberry, Shaimaa Nasr Amin, Reham Shehab El Nemr Esmail, Laila Ahmed Rashed, Maha Mohamed Gamal

**Affiliations:** 1 Department of Medical Physiology, Kasr Al Ainy Faculty of Medicine, Cairo University, Egypt; 2 Department of Pathology, Faculty of Medicine, Fayoum University, Fayoum, Egypt; 3 Department of Biochemistry, Kasr Al Ainy Faculty of Medicine, Cairo University, Egypt

**Keywords:** liver, hepatic encephalopathy, stem cells, hippocampus, basal ganglia

## Abstract

Liver cirrhosis is the outcome of chronic liver injury. The current study aimed to investigate the therapeutic effect of undifferentiated mesenchymal stem cells versus *in vitro *partially differentiated mesenchymal stem cells on liver cirrhosis and hepatic encephalopathy. 50 adult male albino rats constituted the animal model and were divided into the following groups: control, thioacetamide, undifferentiated mesenchymal stem cells and hepatocyte growth factor-differentiated mesenchymal stem cells groups. Cognitive assessment was achieved by open field test and Y-maze task. We measured serum alanine aminotransferase, albumin and transforming growth factor-beta1, gene expression of α-smooth muscle actin, matrix metalloprotein-2, its tissue inhibitor and apoptotic markers: Bax and Bcl2, brain glial fibrillary acidic protein, synaptophysin, and dopaminergic receptors.

## Introduction

Liver cirrhosis represents the final pathologic outcome for the majority of chronic liver diseases (Salama et al., 2014[50]). Due to the functional overcapacity of liver, fibrosis, and even cirrhosis are frequently asymptomatic until complicated by life-threatening events such as variceal hemorrhage, spontaneous bacterial peritonitis and hepatic encephalopathy (HE) (Schuppan & Afdhal, 2008[52]). 

Despite the improvements in the management of liver cirrhosis, the overall outcome of the disease remains unsatisfactory (Bai et al., 2014[3]). Current treatment for liver cirrhosis is to prevent further damage of the functional hepatocytes as well as liver transplantation. Although a significant advance in liver transplantation has taken place, still it has several limitations (Singal and Duchini, 2011[55]). Alternative therapeutic approaches through stem cells may become other options (Chang et al., 2009[14]).

Stem cells are undifferentiated highly specialized cells, having the capacity to renew itself and are capable of dividing to grow different cell types (Ding et al., 2011[17]). Among the various types of stem cells, mesenchymal stem cells (MSCs) in particular have been thought as a promising source of stem cells in regenerative medicine (Shetty et al., 2014[53]) because of their high capability for self-renewal and differentiation without ethical or tumorigenic problems (Jang et al., 2014[26]). However, there are still several challenges including conflicting data regarding MSCs engraftment in the liver and their long-term efficacy, the potential risk of malignant transformation and unwanted mesenchymal lineages differentiation *in vivo* which limit their ability to be used as a mainstream treatment approach for liver regeneration (Volarevic et al., 2014[58]).

Different studies suggested that injured liver tissue secretes various paracrine factors and chemoattractive agents that have a remarkable role in stem cell recruitment, homing process (Lysy et al., 2008[31]). Also, injured liver secretes enhancers of hepatic MSCs differentiation such as hepatocyte growth factor (HGF), fibroblastic growth factor (FGF) (Ishii et al., 2008[25]).

Hepatic encephalopathy (HE) is a common complication in patients with liver cirrhosis. HE not only results in a diminished quality of life but confers a poorer prognosis. The liver cirrhosis in patients without clinical symptoms of HE may show mild cognitive impairment (Romero-Gomez et al., 2007[49]). In severe cases, it can even lead to coma or death with cerebral edema (Poh and Chang, 2012[45]).

Cognitive studies in animal models of HE demonstrated that there is impairment in exploratory behavior (Leke et al., 2012[29]), spatial and non-spatial memory (Nasehi et al., 2013[36]). 

Basal Ganglia (BG) is a key player in a variety of crucial brain functions including reward-based learning, exploratory behavior, action selection, action-gating, motor preparation and timing (Chakravarthy et al., 2010[11]). A common thread that runs through all the roles of the BG is the involvement of dopamine (DA) in regulating the activity of its different nuclei (Kalva et al., 2012[28]).

The aim of the work was to evaluate the therapeutic effect of undifferentiated and hepatocytic *in vitro* partially differentiated mesenchymal stem cells on thioacetamide (TAA) induced liver cirrhosis and HE as a complication as well as their possible therapeutic mechanisms.

## Materials and Methods

The Scientific and Ethical Committee of Physiology Department, Faculty of Medicine, Cairo University approved the experimental procedures, animal handling, sampling, and scarification.

### Experimental animals

50 adult male albino rats weighing 150-200 gram constituted the animal models for this work, housed 2 or 3 per cage and acclimatised for two weeks before the study. We kept all animals under the same environmental conditions at room temperature with free access to water and rat chow all through the work. We used ten rats for isolation of bone marrow-derived MSCs, and divided the remaining 40 animals into the following four groups (10 rats/group):

**Control Group:** healthy male rats (saline injected group)

**TAA Group:** in this group, liver cirrhosis was induced by intraperitoneal injection of 200 mg/kg TAA three times weekly for 12 weeks (Poonkhum et al., 2011[46]) followed by single IV injection of 1 cc phosphate buffer saline

**Undifferentiated MSCs** **Group:** in this group liver cirrhosis was induced as in the TAA group, followed by single IV injection of undifferentiated MSCs in the rats' tail vein (3 million cells in 1 cc phosphate buffer saline/ rat) (Zhang et al., 2010[62])

**Differentiated MSCs Group:** in this group liver cirrhosis was also induced as in the TAA group followed by single IV injection of partially differentiated MSCs in the rats' tail vein (3 million cells in 1 cc phosphate buffer saline/rat) (Zhang et al., 2010[62]).

### Hepatocytic differentiation

**Figure 1:**
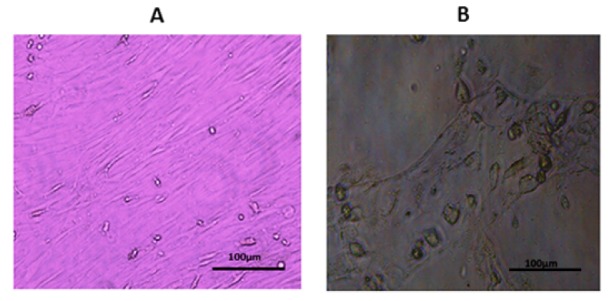
MSC in culture: A: spindle-shaped (undifferentiated); B: rounded shaped (differentiated)

MSCs were induced to differentiate into hepatocyte-like cells using HGF (R&D Systems) and fibroblast growth factor (FGF-4, R&D Systems). Passage 5 cells were cultured in the presence of liver-specific growth factors and were added sequentially (days 0-3: basal medium + 10 ng/mL FGF-4; days 3-6: basal medium + 20 ng/mL HGF; from day 6 on: basal medium 20 + ng/mL HGF, 1×ITS, and 20 µg/L dex). We changed differentiation media every three days. Differentiation was confirmed by morphology (Figure 1(Fig. 1)) and by detection of albumin and α-fetoprotein gene expression in cells as follows:

### PCR detection of human albumin and α-fetoprotein gene expression

Total RNA was extracted from cultured cells using RNeasy purification reagent (Qiagen, Valencia, CA). We generated cDNA from 5 μg of total RNA extracted with 1 μl (20 picomoles) antisense primer and 0.8 μl superscript AMV reverse transcriptase for 60 min at 37 °C. For PCR, we incubated 4 μl cDNA with 30.5 μl water, 4 μl 25 mMMgCl_2_, 1 μl dNTPs (10 mM), 5 μl 10× PCR buffer, 0.5 μl (2.5 U) Taq polymerase and 2.5 μl of each primer containing 10 picomoles. We used the following oligonucleotide primers:

**Albumin** (Forward, 5'-GGCAGGGCT CAGTCAGTAATGA-3'; Reverse, 5'-AGG CCTACCCCAGCCAGTAG-3'),

**α-fetoprotein** (Forward, 5'-TCCTGAAT GGGAGAGGTCC-3'; Reverse, 5'-TCTTGG CCAAAGGAGACG-3'), 

We performed amplification reactions at 94 °C for 30 seconds, 55 °C for 30 seconds, and 72 °C for 60 seconds for 30 cycles.

After 30 days of MSCs injection (Piryaei et al., 2011[44]) we assessed the following parameters:

**I: Behavioral assessment: **we performed cognitive tests twice; before induction of liver cirrhosis and just before scarification; using Y-maze (Arai et al., 2001[2]) and open field tasks (Baykara et al., 2012[5]) to evaluate spatial working memory, locomotion, and anxiety. 

Blood samples were collected from retro-orbital sinuses for assessment of liver functions by measurement of serum albumin, alanine aminotransferase (ALT), ammonia and transforming growth factor beta1 (TGF-β1). Animals then were sacrificed, liver and brain samples obtained for gene expression and histopathological workup.

**II: Biochemical analysis: **Serum ALT and albumin levels were assessed by kits from BioAssay Systems Company (California, USA) using the colorimetric method. Ammonia was enzymatically determined in serum using Ammonia Assay Kit (Sigma-Aldrich, Spruce St., St Louis, USA). Serum TGF-β1 level was assessed by enzyme-linked immunosorbent assay (ELISA) (R&D Systems, Inc., USA) according to manufacturer instructions.

Gene expression of α-SMA, MMP-2, TIMP-2, Bax, Bcl2 in liver tissue and hippocampal glial fibrillary acidic protein (GFAP), synaptophysin and basal ganglia dopamine (D1) receptors by quantitative real-time polymerase chain reaction (qRT-PCR) as follows:

A: RNA extraction and cDNA synthesis

Total RNA was extracted from liver and brain tissue homogenate using SV Total RNA Isolation system *(Promega, Madison, WI, USA)* according to manufacturer's protocol. The yield of total RNA obtained was determined spectrophotometrically at 260 nm. We used the total RNA (0.5-2 μg) for cDNA conversion through cDNA reverse transcription kit (K1621, Fermentas, USA).

B: Real-time PCR

Real-time qPCR amplification and analysis were performed using an Applied Biosystem with software version 3.1 (StepOne™, USA) with the primers shown in Table 1(Tab. 1).

Every reaction consisted of 5 μl cDNA, 1 μl of each primer (400 nM) and 12.5 μl reaction buffers (Platinum SYBR Green) (total reaction volume 25 μl). Real-time PCR cycles consisted of 2 min at 50 °C, for polymerase activation, 40 cycles of 95 °C for 15 s and 60 °C for 1 min and 72 °C for 1 min. We normalised the level of expression of each target gene was about the expression of GAPDH mRNA in that sample using the ΔCt method. Relative differences in gene expression between groups were determined using the comparative Ct (ΔΔCt) method and we calculated fold expression as 2^−ΔΔCt^, where ΔΔCt represents ΔCt values normalised about the mean ΔCt of control samples.

**III: Histological examination and detection of MSCs homing:** Liver and brain tissues were processed and fixed in 10 % formalin solution for 24 hours then embedded in paraffin. The paraffin blocks were serially sectioned at 5 μm thickness then stained with routine hematoxylin-eosin (H&E) for liver and brain and Masson's trichrome (MT) stain (Bancroft, 2008[4]) for assessment of liver fibrosis. We sectioned brains at the levels of hippocampus and basal ganglia. 

Each liver specimen was evaluated for the extent of fibrosis using the criteria of Ishak et al. (1995[24]) and was calculated using Leica Qwin 500 Image Analyzer. Five representative sections were examined for each slide and area percentage was calculated by the image analysis system. The image analysis system consists of a microscope (LEICA DMLB) equipped with a colored digital camera (JVC COLOR VIDEO CAMERA TK- C1380 ½ INCH CCD), linked to a computer via an image-capturing board, having the LEICA software package. We analyzed The captured images through an intelligent interactive image analysis procedure (Leica Qwin 500, version 23, Leica Microsystems Imaging Solutions 1993- 1998) over Microsoft Windows 98, 2^nd^ ed. To capture the widest area of the tissue, we used a 5× magnification objective digitised following interactive light intensity equilibration, and analysed as RGB 24-bit images. The stained section represented fibrosis as blue/green and the parenchyma as red. The total area of the section was the sum of all microscopic fields including parenchyma and fibrosis. We excluded elements of the liver capsule from the computations. 

For detection of cell homing, sections were cut at 4 μm and were examined unstained under the inverted fluorescent microscope to detect cells stained with PKH26 dye to ensure homing and trace the injected cells in liver and brain tissue.

**IV: Statistical analysis:** Data was analysed using the statistical package SPSS version 21. Data was summarised using mean and standard deviation for quantitative variables and frequencies (number of cases) and relative frequencies (percentages) for categorical variables. Comparisons between groups were made using analysis of variance (ANOVA) with multiple comparisons, Bonferroni post hoc test in normally distributed quantitative variables and Kruskal-Wallis test with Mann-Whitney test for non-normally distributed variables (Chan, 2003[13]). 

For comparing categorical data, Chi-square (χ^2^) test was performed (Chan, 2003[12]). P-values less than 0.05 were considered as statistically significant.

## Results

**I: Cognitive and behavioral results: **As shown in Table 2(Tab. 2): The alternation score showed a significant (p<0.05) decrease in the TAA group compared to the control group. After MSCs treatment it increased significantly (p<0.05) only in the differentiated MSCs group compared to the TAA group. However; there is no significant difference between the two MSCs treated groups.

The number of central square entries and center square duration in open field task showed a significant (p<0.05) decrease while the frequencies of freezing, stretch and defecation increased significantly (p<0.05) in the TAA group compared to the control group. Frequencies of stretch and defecation decreased significantly (p<0.05) in the differentiated MSCs group only compared to the TAA group. Although the frequency of grooming didn't increase significantly in the TAA group compared to the control group, yet it decreased significantly (p<0.05) also in the differentiated MSCs group only when compared to the TAA group. These results indicate a state of increased anxiety in the animals belonging to the TAA group, and this anxiety decreased to some extent after MSCs treatment only in the differentiated MSCs group.

**II: Biochemical results:** Table 3(Tab. 3) shows marked deterioration of liver functions in the TAA group in the form of a significant increase in serum ALT level, ammonia and TGF-β1 (p<0.05) and a significant decrease in serum albumin level (p<0.05) when compared to the control. The groups treated with both undifferentiated, and differentiated stem cells showed improvement in the form of significant decrease of serum ALT, ammonia, and the TGF-β1 and significant increase of serum albumin compared to TAA group with better outcome in differentiated than the undifferentiated MSCs group.

Measurement of liver tissue parameters shown in Table 4(Tab. 4) revealed a significant increase in the relative expression of α-SMA after 12 weeks of TAA injection (p<0.05) when compared to the control group. Then it decreased significantly in undifferentiated and differentiated MSCs groups (p<0.05) when compared to the TAA group but it is still significantly (p<0.05) higher than its corresponding value in the control group.

There is a significant decrease in relative expression of MMP-2 (p<0.05) and on the contrary significant increase in relative expression of its inhibitor TIMP-2 (p<0.05) which resulted in significant decrease in the ratio between them (p<0.05) in the TAA group when compared to the control group. This effect opposed after MSCs treatment as denoted by the significant increase in MMP-2 relative expression (p<0.05) and decrease in its inhibitor (p<0.05) in both undifferentiated and differentiated MSCs groups when compared to the TAA group. As a result MMP-2/TIMP-2 ratio also increased in both groups, but this increase was significant only in differentiated MSCs group (p<0.05) and not in the undifferentiated MSCs group when compared to the TAA group.

A significant increase in relative expression of Bax (p<0.05) and a decrease in relative expression of Bcl2 (p<0.05) that led to an increase in the ratio between them (p<0.05) in the TAA group compared to the control group. These results opposed in MSCs treated groups in the form of a significant decrease in relative expression of Bax and Bax/Bcl2 ratio (p<0.05) and a significant increase in relative expression of Bcl2 (p<0.05) compared to the TAA group. In the differentiated MSCs group, relative expression of Bax is significantly lower (p<0.05) and relative expression Bcl2 is significantly higher (p<0.05) than their corresponding values in the undifferentiated MSCs group. On the other hand, results of Bax/Bcl2 ratio show no significant difference between the two MSCs treated groups and also no significant difference between either of them when compared to the control group.

Results of brain tissue parameters are shown in Table 5(Tab. 5). There was a significant decrease (p<0.05) in the relative expression of both synaptic plasticity markers (GFAP and synaptophysin) and basal ganglia dopamine (D1) receptors in the TAA group compared to the control group. There was a significant increase (p<0.05) in the relative expression of the three parameters after MSCs treatment in both differentiated and undifferentiated MSCs groups when compared to the TAA group, without any significant difference between these two MSCs groups. 

**Figure 2:**
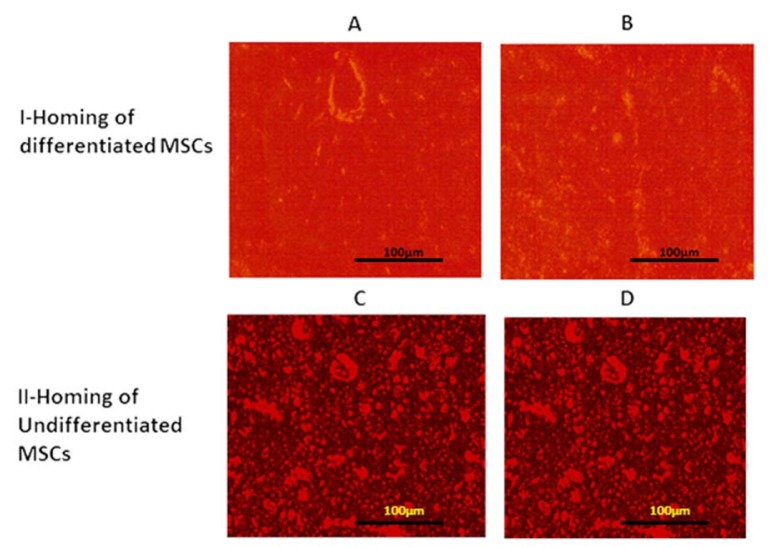
Detection of MCSs homing into liver and brain: I: The presence of red fluorescent indicates homing of differentiated cells into liver (A) and brain (B) tissue; II: The presence of red fluorescent indicate homing of undifferentiated cells into liver (C) and brain (D) tissue.

**III: Detection of MSCs homing: **As seen in Figure 2(Fig. 2) the red fluorescence detected differentiated (I) and undifferentiated (II) MSCs groups in liver (A&C) and brain (B&D) tissues, which indicate homing of PKH26 labelled differentiated and undifferentiated MSCs respectively.

**IV: Histological results: **

A: Liver tissue histopathological examination

**Figure 3:**
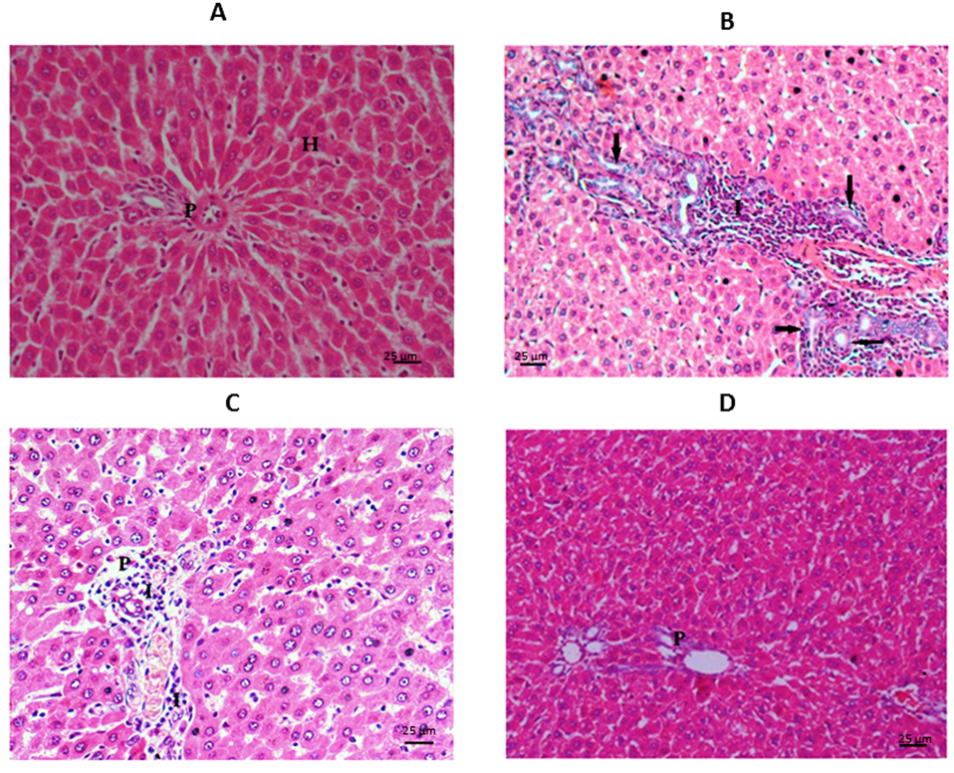
Photomicrograph of the liver sections stained by H&E (X400): A= Control, B= TAA group, C=u ndifferentiated MSCs group, D= differentiated MSCs group

As shown in Figure 3(Fig. 3), in the control group the liver sections have typically appearing hepatocytes (H) running in normal thin trabeculae, portal tract area (P) no fibrosis deposition. TAA group showed fibrous expanded portal areas with septa, early nodular formation, apoptotic hepatocytes, marked inflammatory cell exudates (I), and proliferating bile ducts (arrows) due to the early cirrhotic changes. The undifferentiated MSCs group there was relative in the form of expansion of the portal tract (P) by moderate inflammatory cells (I), the hepatocytes appear irritated with vesicular nuclei. The differentiated MSCs group shows a nearly normal appearance of hepatocytes, with the portal tracts (P) free of fibrosis or inflammation denoting a marked improvement in the differentiated MSCs group.

**Figure 4:**
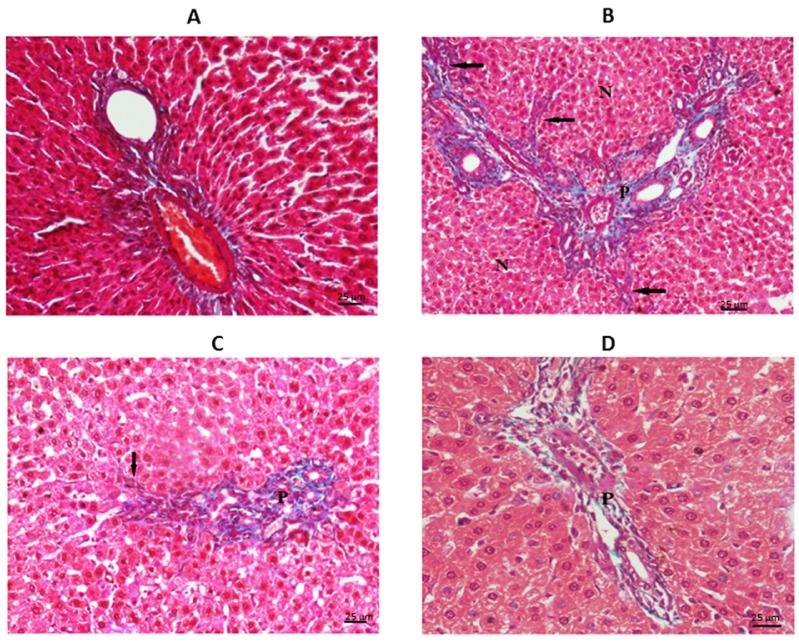
Photomicrograph of the liver sections stained by MT (x400): A= Control, B= TAA group, C= undifferentiated MSCs group, D= differentiated MSCs group

In liver sections stained with MT (Figure 4(Fig. 4)) there is a typical portal tract area containing blood vessel and bile duct with no portal fibrosis in the control group. In TAA group there was expanded portal tracts (P) with excessive fibrous tissue, septa (the arrows), and early nodular formation (N). The undifferentiated MSCs group showed moderate expansion of the portal area (P) by fibrous deposition with few septa formation (the arrows) denoting relative regression of cirrhosis observed in the TAA group after treatment with undifferentiated MSCs.

Furthermore, we measured the % of fibrosis (Table 6(Tab. 6)) that showed a significant increase (p<. 0.05) in the percentage of fibrosis area in the TAA group when compared to the control group. A significant decrease (p<0.05) in the percentage of fibrosis area in MSCs treated groups as compared to the TAA group. This decrease is more prominent in the differentiated MSCs group as the percentage of fibrosis area in this group is significantly lower (p<0.05) than that of the undifferentiated MSCs group. These results were confirmed also by the increase in Ishak score from 0 in the control group to 5 in the TAA group, followed by its regression to 3 and even 1 in the undifferentiated and differentiated MSCs groups respectively.

B: Brain tissue histopathological examination

**Figure 5:**
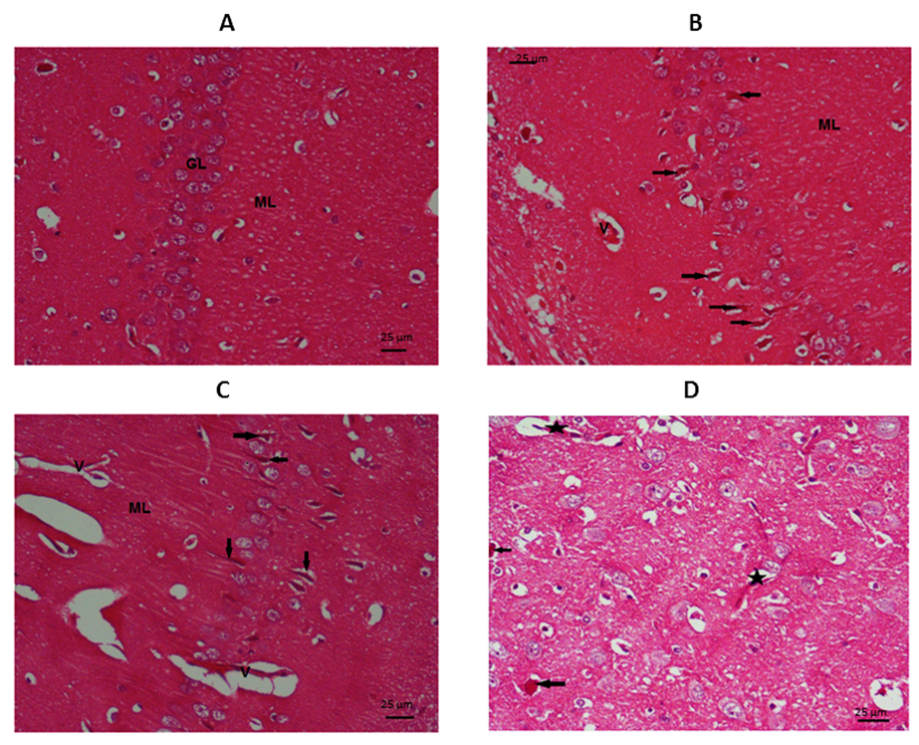
Photomicrograph of the basal ganglia stained by H&E (X400): A= Control, B= TAA group, C= undifferentiated MSCs group, D= differentiated MSCs group

As seen in Figure 5(Fig. 5) basal ganglia in control group showed normal appearance with vesicular nuclei and prominent nucleoli. The neuropil is fibrillary with no clumps or vacuoles. TAA group showed many scattered degenerating ganglion cells (the arrows) in the basal ganglia area, these appear shrunken with dense cytoplasm and inconspicuous nuclei. The neuropil shows areas of increased density, i.e., clumped (C) and foci of vacuolization (V). The undifferentiated MSCs group showed some scattered degenerating ganglion cells (arrows) in the basal ganglia area. They appear shrunken with dense cytoplasm and absent normal vesicular nuclei. The neuropil shows focal clumping (C). In differentiated MSCs group, there are occasional degenerating ganglion cells (arrows), they appear shrunken with dense cytoplasm and inconspicuous nuclei. The neuropil is almost within normal, yet few dilated blood vessel are seen (star).

**Figure 6:**
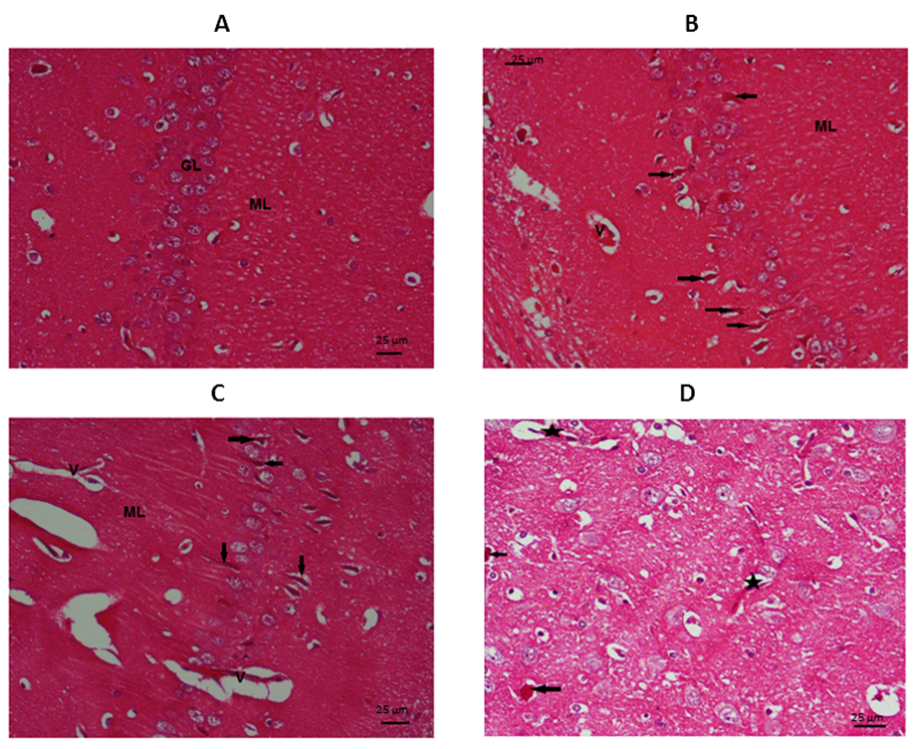
Photomicrograph of granular and molecular layers of the CA1 region of the hippocampus stained by H&E (X400): A= Control, B= TAA group, C= undifferentiated MSCs group, D= differentiated MSCs group

**Figure 7:**
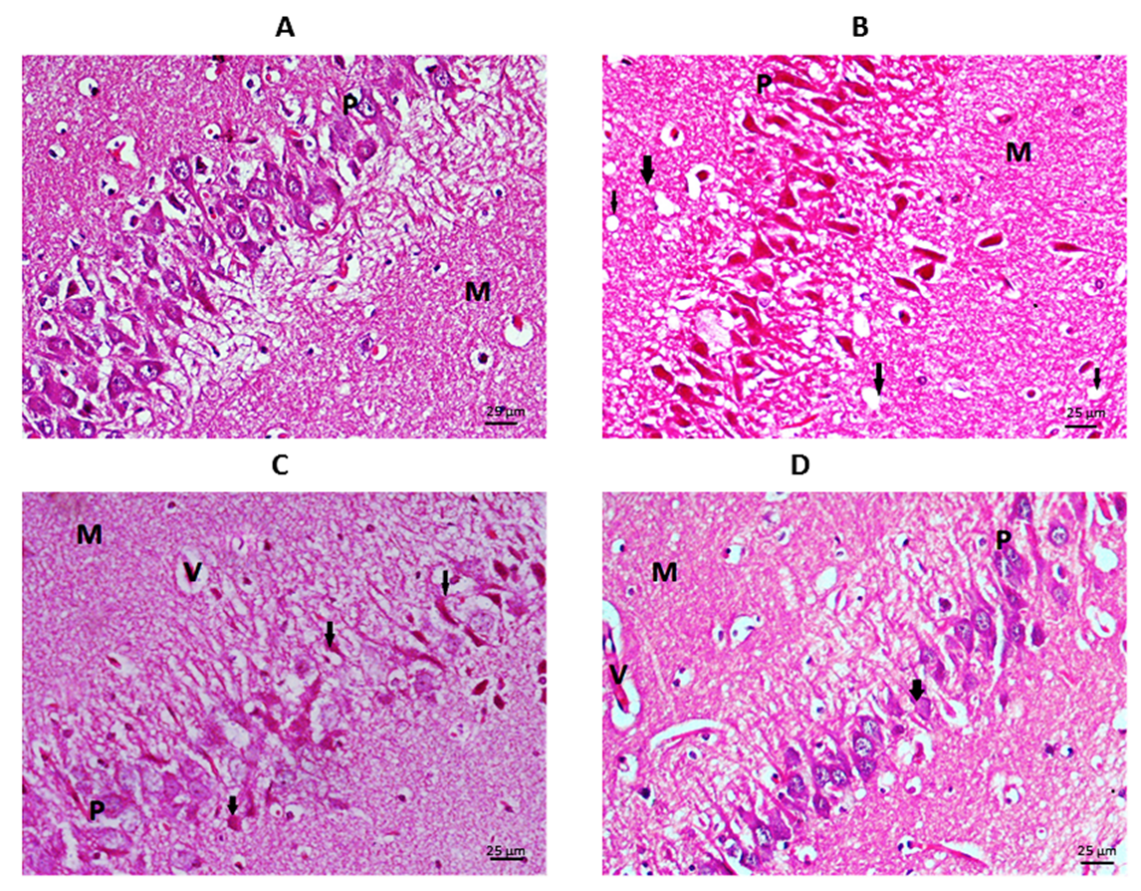
Photomicrograph of the pyramidal layer of the CA1 region of the hippocampus stained by H&E (X400): A= Control, B= TAA group, C= undifferentiated MSCs group, D= differentiated MSCs group

Hippocampal examination (Figures 6(Fig. 6) and 7(Fig. 7)) showed a typical appearance of the neurons of the granular layer of the hippocampus (GL) and the molecular layer (ML) in the control group. The regular arrangement of the pyramidal cells (P) which appear with their vesicular cytoplasm and prominent nuclei and normal dendrites, the molecular layer (M) showed a typical neurofibrillary pattern and vascularity.

TAA group showed many degenerating neurons in the granular cell area (GL), these neurons appear dark and shrunken (the arrows). The molecular layer (ML) shows clumped neuropil which points to decreased connectivity. Congested blood vessels (V) denoting constant irritation and cell injury. There is extensive damage to the pyramidal cells with cell loss and disorganised cells (P), these cells appear degenerated, dark and shrunken. The molecular layer (M) showed vacuolization of the neuropil (arrows) and congested blood vessels (V).

The undifferentiated MSCs group showed the persistence of some injury in the granular cells area (GL) of the hippocampus in the form of multiple congested blood vessels (V) denoting increased blood flow. Also, the scattered degenerating neurons (arrows) which appear dark shrunken with focally vacuolated cytoplasm (less than in the TAA group). The molecular layer (ML) showed moderately thickened neuropil with some micro-cystifications pointing to decreased connectivity. There is scattered degenerating pyramidal neurons (arrows) appearing dark and shrunken, in contrast to the normal adjacent neurons (P) which have vesicular nuclei and prominent nuclei. The molecular layer (M) showed fine neuropil and congested blood vessels (V).

The differentiated MSCs group showed the granular cell area of the hippocampus (GL) with only occasional degenerating neurons (arrows) which appear dark and shrunken in contrast to the normal neurons which have vesicular nuclei. The molecular layer (ML) contains few congested blood vessels (V) and mildly thickened neuropil with mild clumping.

## Discussion

MSCs have gained popularity for their potential as seed cells to treat various human diseases (Xu et al., 2012[60]). *In vitro* hepatic differentiation of MSCs followed by their *in vivo* inoculation also proved its success in improving liver fibrosis in animal models (Zhao et al., 2012[63]).

TAA is known hepatotoxic, which produces hepatic necrosis and cirrhosis by oxidative stress mediated acute hepatitis and induces apoptosis of hepatocytes in the liver (Sun et al., 2000[56]). We demonstrated hepatocytic apoptosis indicated by significant increase in relative expression of pro-apoptotic marker Bax and a decrease in relative expression of anti-apoptotic marker Bcl2 which led finally to an increase in the ratio between them (Bax/Bcl2). Contradictory to our results, Salama et al. (2013[51]) showed no significant difference of Bax between the cirrhosis group and the control group while the level of anti-apoptotic Bcl-2 showed a significant increase in the cirrhosis group compared with the control group and a significant decrease in the ratio between them in the cirrhosis group. Our histopathological results after TAA administration are consistent with Elhaggagy et al. (2014[20]). 

HSC activation is considered the pivotal event in the development of liver cirrhosis (Lin et al., 2014[30]) being the primary source of ECM (Hernandez-Gea and Friedman, 2011[21]). When activated, HSCs differentiate into α-SMA expressing myofibroblast-like cells capable of depositing ECM (Puche et al., 2013[47]). In our study, HSC activation was confirmed by the significant increase in relative expression of α-SMA in liver tissue of TAA group when compared to the control group. These results are in agreement with those of Elhaggagy et al. (2014[20]) who assessed α-SMA immunohistochemically and found that it increased significantly in cirrhotic rats compared to healthy ones.

The current study revealed a significant increase in serum TGF-β1 in TAA treated rats compared to control rats. This increase can be related to the TAA-induced oxidative action that directly activates HSCs converting them to myofibroblasts and indirectly causes damage and apoptosis of hepatocytes. The damaged hepatocytes activate KCs that together with apoptotic hepatocytes release TGF-β1; the major fibrogenic cytokine which is critical for activation of HSCs. Moreover; TGF-β1 together with deposited ECM have an antiproliferative and pro-apoptotic effect on hepatocytes (Swaroop and Gowda, 2012[57]).

Progression of liver fibrosis depends on the rate of deposition and degradation of ECM which is controlled by MMPs and their tissue inhibitors (TIMPs). The balance between MMPs and TIMPs controls progression or regression of liver fibrosis (Park et al., 2010[41]). This balance is qualified in experimental studies by expressing it in the form of a ratio between MMP and its tissue inhibitor TIMP (MMP/TIMP) (Yang et al., 2011[61]).

The current work showed a significant decrease in relative expression of MMP-2 while the relative expression of TIMP-2 showed a significant increase in the TAA group compared to the control group. This increase in TIMP-2 and the decline in MMP-2 caused a marked drop in the ratio between them (MMP-2/TIMP-2). Our results agree with previous studies indicating that chronic liver damage usually favors fibrogenesis over fibrolysis (Park et al., 2010[41]). In agreement with our results, the work done by Madro et al. (2012[32]) showed a significant decrease of serum MMP2 activity in stages B and C of alcoholic cirrhosis patients rather than early stages. HE is a serious complication of a fibrotic liver. 

HE is related to an accumulation of toxic metabolites in the brain, especially ammonia (Albrecht and Zielińska, 2014[1]). TAA may induce encephalopathy by both direct and indirect mechanisms. Indirect mechanisms are mediated primarily by liver failure induced hyperammonemia that causes brain edema, alterations in neurotransmission, oxidative stress, mitochondrial dysfunction and neuronal death (Mladenović et al., 2012[33]).

TAA-induced hyperammonemia is evidenced in the current study, as there is a highly significant increase in serum level of ammonia in the TAA group compared to the control group. Our findings agree with the results obtained by Jayakumar et al. (2014[27]) in which induction of liver cirrhosis and consequently chronic HE by TAA resulted in significant elevation of blood and brain ammonia. 

In the current study behavioral evaluation of our experimental animals by Y-maze and open field tasks revealed marked deterioration of the spatial memory and observable increased anxiety in the TAA group compared to the control. The work done by Hosseini et al. (2013[22]) was consistent with ours, as it denoted significant deterioration of spatial memory with slight, insignificant impairment of locomotor activity as assessed by open field task.

Astrocytes are the primary neural cells involved in this pathogenesis of hyperammonemia induced HE (Jayakumar et al., 2014[27]). Astrocytic swelling may result from altered levels of expression of genes coding for proteins implicated in cell volume regulation including GFAP. GFAP is a major protein of cytoskeletal network resulting in impairment of visco-elastic properties of astrocytes facilitating their swelling (Bélanger et al., 2002[7]). GFAP is also considered a parameter of astrocytic activity (Bernardi et al., 2013[9]). In the current work, we found a significant decrease in the relative expression of hippocampal GFAP in the TAA group that denotes astrocytic swelling and dysfunction which most probably was one of the reasons for the deterioration of cognitive and motor performance in this group. 

The hippocampus is brain region that critically is involved in the acquisition, performance of spatial and non-spatial memory tasks (Bouton and Moody, 2004[10]). In this study, examination of CA1 area of the hippocampus in the TAA group revealed degenerating neurons in the granular cell area, vacuolization and clumped neuropil which points to decreased connectivity in the molecular layer and extensive damage to pyramidal cells with cell loss and disorganised cells in the pyramidal layer. This finding is in agreement with studies by Peeling et al. (1993[42]) and Norton et al. (1997[37]).

Synaptophysin is an abundant integral membrane protein of pre-synaptic vesicles involved in the regulation of neurotransmitter release and synaptic plasticity (Jayakumar et al., 2014[27]). In this study, there was a significant decrease in relative expression of hippocampal synaptophysin after induction of liver cirrhosis. This reduction of synaptophysin expression most probably resulted in the behavioral changes which occurred in the TAA group. Our finding is concomitant with the work done by Jayakumar et al. (2014[27]). 

Although the hippocampal glutaminergic system plays a critical role in synaptic plasticity (Bear and Malenka, 1994[6]), a significant involvement of dopamine in synaptic plasticity has also been demonstrated. Although multiple dopamine receptor subtypes contribute to different aspects of learning and memory, the D1 receptors seem to play a more prominent role (El-Ghundi et al., 2007[19]). 

Our results revealed a significant reduction of basal ganglia D1 receptors in the TAA group, which might be one of the mechanisms by which spatial memory deterioration took place. The reduction of D1 receptors expression in the current work may be due to hyperammonemia which resulted from liver cirrhosis and caused NMDA receptors loss. We may propose that selective loss of NMDA receptors caused decreased relative expression of D1 receptors due to the interaction between dopaminergic and glutaminergic systems (Pei et al., 2004[43]).

Results of the current study also demonstrated a significant increase in anxiogenic-like behaviors in rats belonging to the TAA group compared to the control group. Dopaminergic systems are shown to play pivotal roles in the regulation of anxiety-like behaviors (Reza Zarrindast et al., 2013[48]). Conversely, we can conclude that dysfunctional dopaminergic system due to either decreased expression of DA receptors or decreased DA itself most probably leads to increased anxiety level. 

MSCs were administered as a single bolus injection of 3 million cells/rat following TAA administration for 12 weeks. Homing of MSCs was confirmed one month after their injection by examination of liver and brain tissue under the inverted fluorescent microscope and detection of red fluorescence of PKH26 labelled cells.

Homing and liver repopulation following MSCs transplantation triggered by liver damage. Different studies suggested that injured liver tissue secretes various paracrine factors and chemoattractive agents that regulate homing process (Dalakas et al., 2005[16]). Some of these growth factors released from damaged liver cells may also play a role in the enhancement of hepatic differentiation of MSCs including HGF (Ishii et al., 2008[25]) and FGF (Berg et al., 2007[8]).

The results of our study revealed that both undifferentiated and partially hepatocytic differentiated MSC therapies elicited pronounced regression of liver cirrhosis and improvement in all studied parameters. This improvement was more marked in the differentiated MSCs group.

In the undifferentiated MSCs group, histopathological results revealed relative regression of cirrhosis and improved liver cell injury while in the partially differentiated MSCs group, regression of cirrhosis was much clearer. This improvement was confirmed by significant regression of fibrosis staging and percentage of fibrosis areas especially in the differentiated MSCs group when compared to the TAA group. These histopathological findings were concomitant with profound improvement in liver functions following MSCs treatment, especially in differentiated MSCs group when compared with TAA group.

In an attempt to explain our results, we tried to analyse the impact of MSC therapy on the primary factors contributing to fibrogenesis and the interaction between them. Hypotheses about the mechanism by which MSCs may contribute to liver regeneration in our study have included paracrine effects and transdifferentiation of MSCs into hepatocytes (Parekkadan et al., 2007[39]).

The effect of MSCs on liver cirrhosis could be achieved by their inhibition of HSC activity and proliferation and downregulation of collagen gene expression (Parekkadan et al., 2007[40]) and induce their apoptosis via a paracrine mechanism (Hwang et al., 2012[23]). This effect is mediated through secretion of variable growth factors including HGF (Wang et al., 2009[59]). HGF is a potent anti-apoptotic mitotic promoter for hepatocytes, apoptotic promoter for HSCs (Chen et al., 2013[15]). It can also suppress HSC activity through downregulation of TGF-β1 (El-Ansary et al., 2012[18]) and can reduce gene expression of collagen type I and type III. 

In the current study there was a significant reduction in relative expression of α-SMA in both MSCs treated groups and a significant decrease in serum TGF-β1 after MSCs treatment which was more prominent in the differentiated MSCs group. This decline in serum TGF-β1 may be attributed to the elimination of activated HSCs (Mormone et al., 2011[35]) mediated by secretion of HGF from the transplanted MSCs (El-Ansary et al., 2012[18]).

We demonstrated a significant increase in MMP-2 gene expression following MSC therapy. On the contrary, TIMP-2 gene expression showed a significant decrease in both MSC-treated groups when compared to TAA group, which was more marked in the differentiated MSCs group. Increased MMP-2 and decreased TIMP-2 led to an increase in the MMP-2/TIMP-2 ratio in MSC-treated groups. This increase may explain the regression of liver fibrosis after MSC transplantation in our study with augmentation of ECM degradation. We confirmed these results by the marked regression of fibrosis stage in both MSC-treated groups in comparison with TAA groups especially the differentiated MSCs group in which cirrhosis regression was almost complete. This improvement clearly reflected in histopathological findings which revealed nearly normalised hepatic architecture in the differentiated MSCs group.

Our results are consistent with studies by Zhao et al. (2012[63]) who showed that bone marrow-derived MSCs were able to suppress CCl_4 _induced liver fibrosis through the high expression of MMP-2 and MMP-9. On the other hand, downregulation of TIMP-2 could be due to apoptosis and arrested activation and proliferation of HSCs by MSCs (as HSCs are its only source).

Our results demonstrated a marked reduction in the relative expression of pro-apoptotic marker Bax and a significant increase in relative expression of anti-apoptotic marker Bcl2 following MSCs treatment. It was reported that MSCs secrete agonists that inhibit hepatocyte apoptosis (Mohsin et al., 2011[34]).

MSCs also have multiple immunomodulatory effects. They can counteract inflammation and inhibit the proliferation and function of the major immune cell populations, including T cells, B cells and NKCs (Shi et al., 2011[54]). This anti-inflammatory action may explain the decreased necro-inflammatory reaction in MSC-treated groups demonstrated by our histopathological results.

Our study showed the better outcome of partially differentiated MSCs to regress liver cirrhosis compared to undifferentiated MSCs. This impact was evident from the difference in histopathological results, which were almost normal and liver functions particularly serum albumin level, that was significantly higher in the differentiated MSCs group compared to the undifferentiated MSCs group. In agreement with our work, Oyagi et al. (2006[38]) found that the transplantation of HGF treated MSCs markedly improved liver fibrosis in rats when compared with undifferentiated MSCs. 

Concerning HE, our results revealed an improvement in spatial memory after MSCs treatment which was more observable in the differentiated MSCs group. Also, there was a relative decrease in anxiogenic behavior in differentiated MSCs group but not in the undifferentiated MSCs group. We supported our results by the histopathological findings. In the undifferentiated MSCs group, the hippocampus shows the persistence of some features of the injury. On the other hand, differentiated MSCs group showed relatively clearer signs of brain injury regression.

Regression of liver cirrhosis after MSCs administration was mostly the cause of improvement of cognitive function in both MSCs groups. Liver cirrhosis induced hyperammonemia has the pivotal role in the neurotoxic effects of TAA (Mladenović et al., 2012[33]), and we demonstrated that it is most probably the main cause of behavioral changes in the TAA group. Therefore, we suggest that the significant drop in serum ammonia level following MSCs treatment is the cause of improvement in cognitive functions in the two MSCs groups. We suppose that the marked decrease in serum ammonia level decreased astrocytic swelling and restoration of astrocytic function which revealed the significant increase in relative expression of GFAP after MSCs administration. Since our results showed that both types of MSCs undergone homing in the brain, even to a small extent, MSCs might have a direct role in the improvement of HE symptoms. Another proposed reason for improvement of spatial memory and anxiety after MSCs treatment is the significant increase in relative expression of D1 receptors in both MSCs groups when compared to TAA group.

It is worth noting that although relative expression of GFAP, synaptophysin, and D1 receptors increased significantly in both MSCs groups to nearly the same extent, spatial memory improvement was more prominent in the differentiated MSCs group compared to the undifferentiated MSCs group. Also, anxiety symptoms were observed to be improved only in the differentiated MSCs group which needs further investigation.

In conclusion, MSCs therapy could regenerate cirrhotic liver, improve liver function and hepatic encephalopathy (HE) with hepatogenic partially differentiated MSCs are more efficient than undifferentiated MSCs. The superiority of partially differentiated MSCs is most probably due to augmented homing and differentiation abilities of these cells. 

## Conflict of interest

There is no conflict of interest.

## Author contributions

The experiments have been performed at Department of Medical Physiology, Kasr Al Ainy Faculty of Medicine, Cairo University, Department of Biochemistry Kasr Al Ainy Faculty of Medicine, Cairo University and Department of Pathology, National Research Institute. The authors contributed to the work as following:

*Dalia Azmy Elberry: *Performed the experimental part, analysed the data and shared in manuscript writing

*Shaimaa Nasr Amin: *Design and follow up of the work, demonstration of the behavioral tests, shared in manuscript writing

*Reham Shehab El Nemr Esmail:* Pathological work and shared in manuscript writing

*Laila Ahmed Rashed: *Biochemical work and shared in manuscript writing

*Maha Mohamed Gamal: *Design and follow up of the work, shared in manuscript writing.

All authors approved the final version of the manuscript and agreed to be accountable for all aspects of the work in ensuring that questions related to the accuracy or integrity of any part of the work are appropriately investigated and resolved.

## Funding

Cairo University partially funded this work.

## Acknowledgements

We would like to thank technicians at the Physiology Department for their kind help in the study.

## Figures and Tables

**Table 1:**
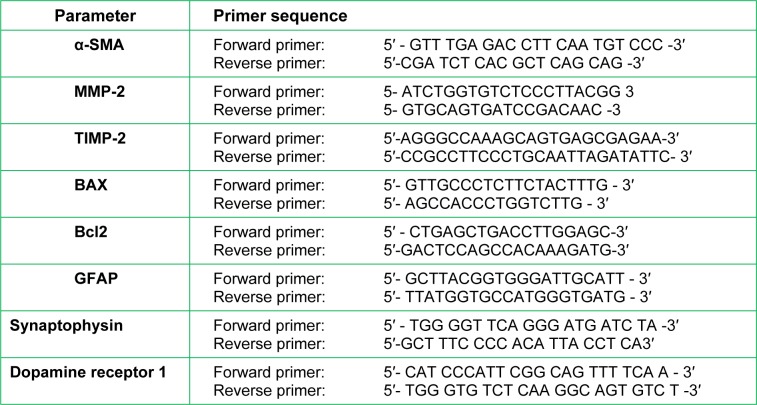
The oligonucleotide primers sequence of the studied genes (*in vivo*)

**Table 2:**
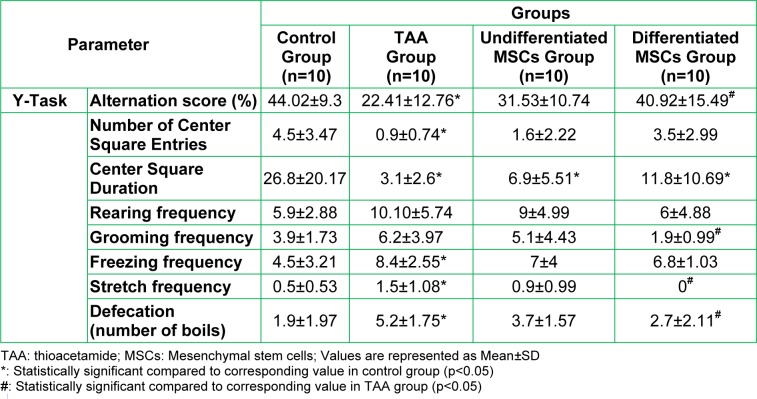
Comparison of the performance in Y-Maze and open field tests in the studied groups at the end of the work

**Table 3:**
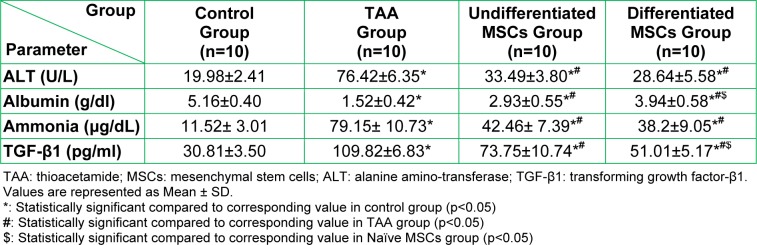
Serum markers of liver function in the studied groups

**Table 4:**
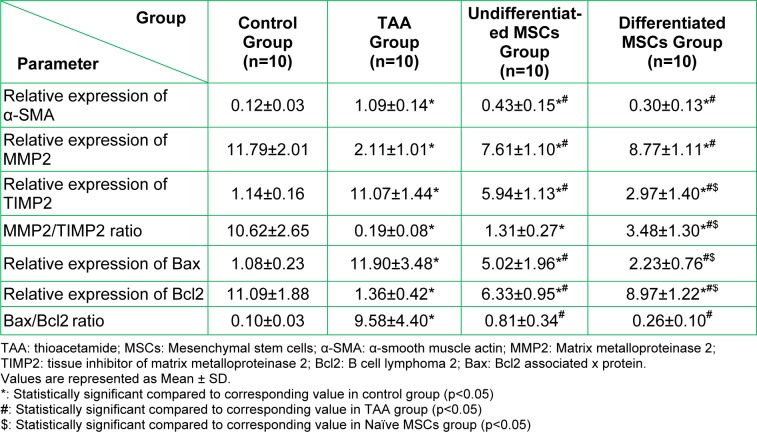
Measured hepatic parameters by gene expression in the studied groups

**Table 5:**
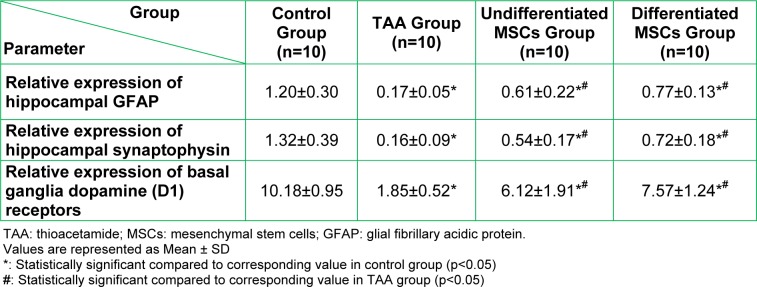
Measured brain parameters by gene expression in the studied groups

**Table 6:**
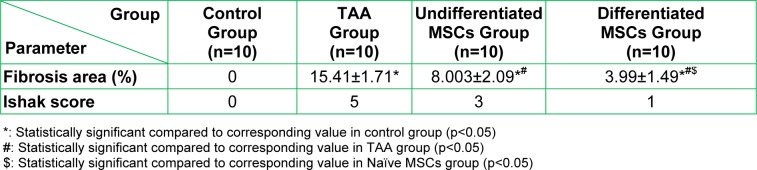
Percentage of fibrosis area in liver of the studied groups and Ishak score of each group
